# Clinical Implications of TβRII Expression in Breast Cancer

**DOI:** 10.1371/journal.pone.0141412

**Published:** 2015-11-09

**Authors:** Ningning Gao, Qixi Zhai, Yinyan Li, Kun Huang, Donglin Bian, Xuemei Wang, Caigang Liu, Hong Xu, Teng Zhang

**Affiliations:** 1 Ultrasonic Diagnosis Department, First Hospital of China Medical University, Shenyang, Liaoning Province, China; 2 Department of Breast Surgery, Second Hospital of Dalian Medical University, Dalian, Liaoning Province, China; 3 Department of Breast Surgery, Liaoning Province Cancer Hospital and Institute, Shenyang, Liaoning Province, China; The University of Texas MD Anderson Cancer Center, UNITED STATES

## Abstract

**Objective:**

To explore the relationship between TβRII [type II TGFβ (transforming growth factor β) receptor] expression and clinicopathological characteristics, and to evaluate the prognostic significance of TβRII expression in breast cancer.

**Methods:**

Clinicopathological data and prognostic information of 108 patients with histologically confirmed breast cancer who were surgically treated at China Medical University between January 2007 and September 2008 were reviewed and the association between the clinicopathological characteristics and TβRII expression was analyzed by chi-square test and multivariate analysis. The expression of TβRII was assessed by immunohistochemistry.

**Results:**

Of the 108 patients, 60 cases were TβRII positive and 48 cases were negative. There was no significant association between TβRII expression of the patients older than 40 years and that of the younger than 40 years (56.0% *vs* 50.0%; *P* = 0.742). The TβRII expression rate was significantly increased in patients with lymph node metastasis compared to those without lymph node metastasis (67.40% *vs* 46.8%; *P* = 0.033). Statistically significant relationships were found between increasing tumor clinical stage and high TβRII expression (*P* = 0.011). TβRII expression was not associated with the expression of ER(estrogen receptor)、PR, (progesterone receptor)、Her-2 (human epidermal growth factor receptor 2) (*P* = 0.925,*P* = 0.861, and *P* = 0.840, respectively). Patients with high TβRII expression showed poorer 5-year disease-free survival (DFS) compared to those with low expression (66.7% *vs* 45.6%; *P* = 0.028) by univariate analysis. Survival analysis demonstrated that TβRII was associated with poor DFS (*P* = 0.011). Subgroup analysis revealed that TβRII expression was associated with shorter DFS in patients with lymph node metastasis, ER-positive, PR-positive or Her-2-negative tumors (*P* = 0.006, *P* = 0.016, *P* = 0.022, and *P* = 0.033, respectively). Cox regression analysis revealed that high TβRII expression was related to poor 5-year DFS, and it was an independent factor for predicting the poor outcome for breast cancer patients (*P* = 0.016).

**Conclusions:**

High levels of TβRII expression were associated with lymph node metastasis, increasing tumor clinical stage, and poorer 5-year DFS in patients with breast cancer. TβRII may be a potential prognostic marker for breast cancer.

## Introduction

Breast cancer accounts for 23% (1.38 million) of all new cancer cases and 14% (458,400) of all deaths by cancer [1]. At present, breast cancer is the most common malignancy in women and the leading cause of death for cancer among females globally [2]. Although various breast cancer treatments including surgery, radiotherapy, endocrine therapy, trastuzumab, cytotoxic therapy, and other biological agent therapies have improved patient survival in recent years, the overwhelming majority of deaths due to cancer are because of recurrence and metastasis. Many studies have revealed that TGFβ (transforming growth factor β) signaling plays a major role in cancer metastasis, and regulation of TGFβ signaling is important for breast cancer therapy [3,4].

The TGFβ superfamily is a large, evolutionarily conserved family of secreted multifunctional peptides [5], composed of 33 structurally similar cytokines (bone morphogenic proteins, activins, and TGF-β ligands), which are important factors in developmental biology, including mammary gland development [6]. TGFβ signaling has tumor-suppressive and tumorigenic effects in accordance with tumor stage [7,8]. The effects of TGFβ are mediated by three TGFβ ligands, TGFβ1, TGFβ2 and TGFβ3 through binding to a heteromeric complex of transmembrane TGFβ serine/threonine kinase type I and type II receptors (TβRI and TβRII). TβRII [type II TGFβ receptor] is crucial for the regulation of TGFβ signaling in tumor initiation, progression, and metastasis [9].

TβRII is the specific receptor for TGFβ ligands. TβRII, also known as TGFβR2 (TGFβ receptor type-2), is a 567 amino acid single-pass type I membrane protein that contains one protein kinase domain. Gobbi et al. showed that downregulated TβRII was associated with an increased risk of developing invasive breast cancer, and the absence of TβRII correlated with high-grade human carcinoma in situ and invasive breast cancer [7,10]. Previous studies revealed that the complete loss of TβRII tissue expression in breast cancers was associated with the development of distant metastases and poor overall survival, and absence of TβRII in carcinoma cells promotes mammary tumor growth [9,11]. *TβRII* can act as a tumorsuppressor gene [12,13], and decreased expression in other cancers such as head and neck squamous cell carcinoma is related to aggressive cellular behavior [11,14–17]. Moreover, Chen et al. reported that decreased TβRII more common in non-small cell lung cancer patients with lymph node metastasis and increasing pathological stage [18]. However, analysis of clinical tumor samples has demonstrated that breast cancer patients with high TβRII expression have poor progression-free survival [19,20]. Takanami et al. found that the presence of immunoreactivity for TβRI and/or TβRII is correlated with poor prognosis in lung adenocarcinoma [21].

These paradoxical findings support the notion that TGFβ functions as a tumor suppressor or a tumor promoter in cancer development. In the present study, TβRII expression was evaluated to analyze its relationship with clinicopathological features and prognosis in a cohort of 108 breast cancer patients.

## Materials and Methods

### Patients

A total of 108 patients with histologically confirmed breast cancer who were surgically treated at China Medical University between January 2006 and September 2007 were included in this study. The mean age was 51.26 years (range, 33–75years). Lymph node metastasis was present in 46 patients. The patients were classified into clinical stages I (n = 30), II (n = 64) and III (n = 14), according to the TNM staging system. The criteria to include a patient in this study were as follows: (a) curative operations were performed; (b) resected specimens were pathologically examined; (c) more than 10 lymph nodes were pathologically examined after the operation; and (d) a complete medical record was available including pathologic tumor size, lymph node status, tumor clinical stage and biomarkers status (ER, PR and HER-2). In addition, follow-up data was available for 108 patients, who were followed-up from 5 to 75 months (median, 54.96 months; mean 61.00 months). The study protocol was approved by the Ethics Committee of China Medical University. Written informed consent was obtained from all the participants involved in the study.

### Tissue specimens

Thin slices of tumor tissue of all cases received in our histopathology unit were fixed in 4% formaldehyde solution (pH 7.0) for periods not exceeding 24h. The tissues were processed routinely for paraffin embedding and 4μm-thick sections were cut and placed on glass slides coated with 3-aminopropyl triethoxysilane for immunohistochemistry.

### Immunohistochemistry

Rabbit-anti-human monoclonal antibody against TβRII (sc-400; Santa Cruz) and Ready-to-SP (streptavidin-peroxidase) immunohistochemical detection kit (SP-9001; Santa Cruz) were used in this study. The sections were deparaffinized in xylene (I, II, and III) for 15 min each, and rehydrated with graded ethanol solutions for 35 min. Endogenous peroxidase was blocked by incubating the sections in 3% hydrogen peroxide methanol for 10 min. Heatinduced antigen retrieval at 100°C for 2.5 min was performed in 10 mmol/l citrate buffer solution (pH 6.0) in a pressure cooker. After blocking nonspecific reactivity with 10% normal goat serum for 15 min at room temperature, sections were incubated overnight at 4°C with a primary rabbit-anti-human antibody against TβRII (1:500) followed by 15 min incubation at 37°C with a secondary goat-anti-rabbit antibody. Negative control was prepared by substituting the primary antibody with PBS. The samples were subsequently treated with the streptavidin biotin complex for 15 min. Staining was visualized using a diaminobezidine solution, and sections were counterstained with hematoxylin, dehydrated, and cover-slipped with mounting medium.

### Evaluation of immunostaining

The presence of brown-yellow particles on the cell nucleus following the immunohistochemical assay indicated positively-stained cells, and the degree of the staining was semi-quantitatively classified according to the percentage of positive cells. The specimens were labeled with (-) if they contained <5% positive cells, (+) for 5-20% positive cells, and (++) for >20% positive cells. We considered specimens graded as ++ to be TβRII positive.

### Statistical analysis

SPSS statistics software (Version 17.0, SPSS Inc., Chicago, IL, USA) was used for all statistical analyses. The association between TβRII expression and the clinicopathological characteristics were analyzed using the Chi-squared test. Five-year disease-free survival (DFS) was showed as the number of months from surgery to the occurrence of an event (distant metastasis or disease-related death). Survival curves were constructed using the Kaplan-Meier method, and the survival comparison was examined using the log-rank test. Multivariate analysis for DFS was carried out using Cox proportional hazards model,where some potential prognostic factors were included. All tests were two-tailed, with a confidence interval of 95%. A *P*-value of less than 0.05 was considered to indicate a statistically significant difference.

## Results

### TβRII expression

**Fig 1:**
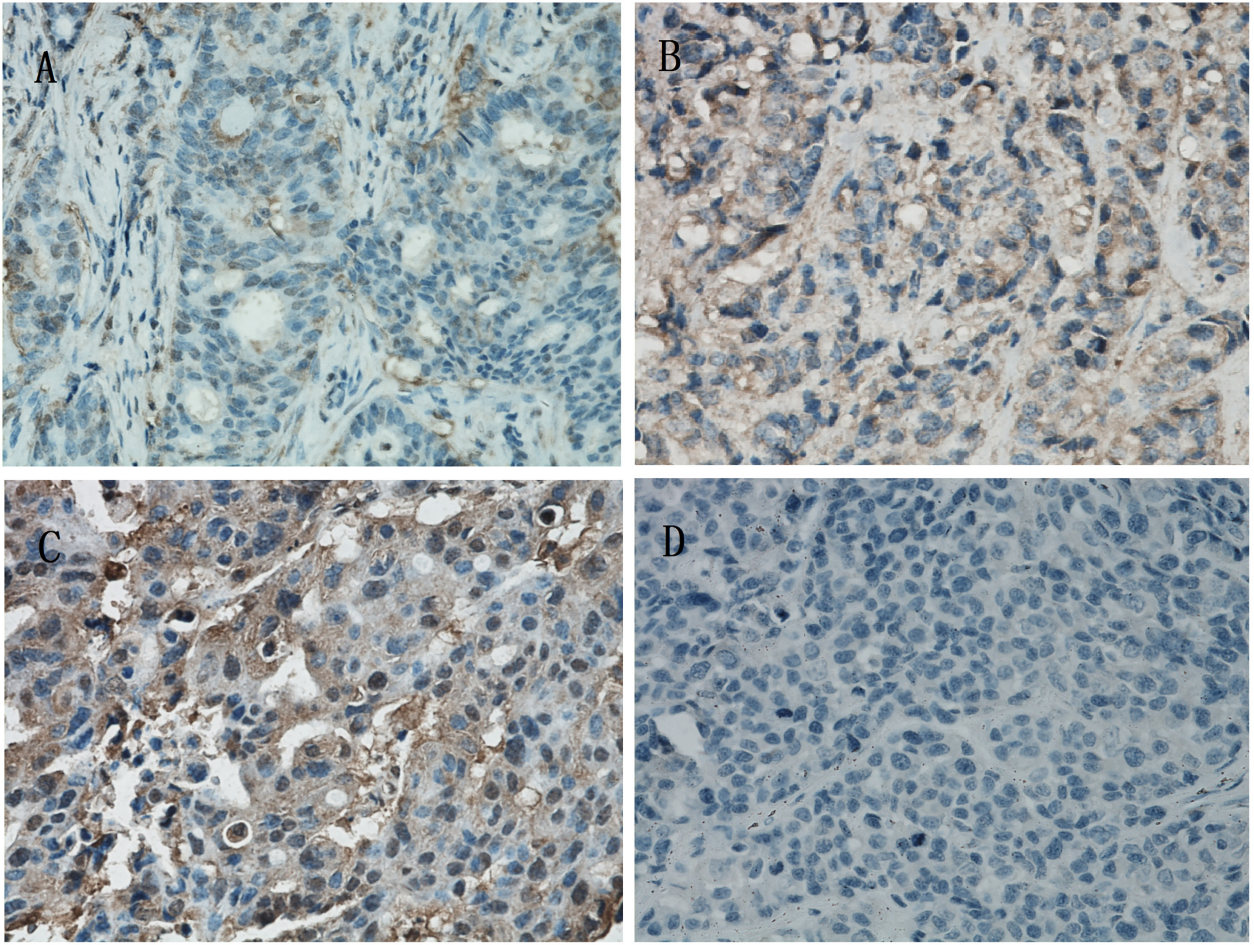
Immunohistochemical staining of TβRII (transforming growth factor-βreceptor type II) protein expression in breast cancer cells. SP staining; Magnification, ×400. (A) TβRII expression was labeled as (-). (B) and (C) TβRII protein expression in the two images were labeled as (+) and (++) respectively. (D) The negative controls showed no TβRII expression.

Immunohistochemical detection showed that TβRII was negatively expressed and was labled as (-) (Fig 1A). The TβRII was positively expressed and was labeled as (+) and (++) respectively (Fig 1B and 1C). There was no TβRII expression in negative controls (Fig 1D). As shown in Table 1, 60 tumors (55.5%) displayed positive expression of TβRII and 48 displayed negative expression (44.5%). In patients with tumor size lager than 2.0cm, the percentage of TβRII expression was 50.87% and in the patients with tumor size between 2 cm and 5 cm, the percentage was 60.78%. In the 14 cases classified as tumor stage III, there were 13 cases with positive TβRII expression (92.86%), and in the 94 cases with tumor stage lower than II, there were 47 cases with TβRII expression (50.0%). The percentage of lymph node-positive cases was 67.39% in patients with positive TβRII expression. In this study, in patients with ER-negative, PR-negative, and HER2-negative disease, the percentage of TβRII expression was 56.3%, 54.55%, and 56.10%, respectively.

**Table 1:** Relationships between TβRII and clinicapathological feathers.

Varible	n	TβRII^a^ ^-^	TβRII ^+^	*P*-value
Age				
>40	98	43	55	0.742
≤40	10	5	5	
Tumor size				
≤2cm	57	28	29	0.301
2-5cm	51	20	31	
Nodes				
Nagative	62	33	29	0.033
Positive	46	15	31	
Clinial stage				
I	30	15	15	
II	64	32	32	0.011
III	14	1	13	

^a^TβRII,typeIITGFβ(transforming growth factorβ)receptor

### Relationships between TβRII expression and clinicopathological features

As shown in Table 1, TβRII expression was not related to age or tumor size (*P* = 0.742, and *P* = 0.301, respectively). The expression levels of TβRII in patients with lymph node metastasis were significantly higher compared with patients without lymph node metastasis, and a statistically significant difference was observed between the two groups (*P* = 0.033). Statistically significant relationships were found between increasing tumor stage and high TβRII expression (*P* = 0.011).

### Relationships between TβRII expression and ER,PR,and Her-2

As shown in Table 2, the associations between the expression of TβRII and ER, PR, and Her-2 were not found to be significantly different (*P* = 0.925, *P* = 0.84, and *P* = 0.861, respectively).

**Table 2:** Relationships between TβRII and ER, PR, Her-2.

Variable	n	TβRII ^-^	TβRII ^+^	*P*-value
ER^a^				
Nagative	32	14	18	0.925
Positive	76	34	42	
PR^b^				
Nagative	44	20	24	0.84
Positive	64	28	36	
Her-2^c^				
Nagative	82	36	46	0.861
Positive	26	12	14	

^a^ER,estrogen receptor.

^b^PR, progesterone receptor.

^c^Her-2,human epidermal growth factor receptor 2.

### Survival outcome

**Fig 2:**
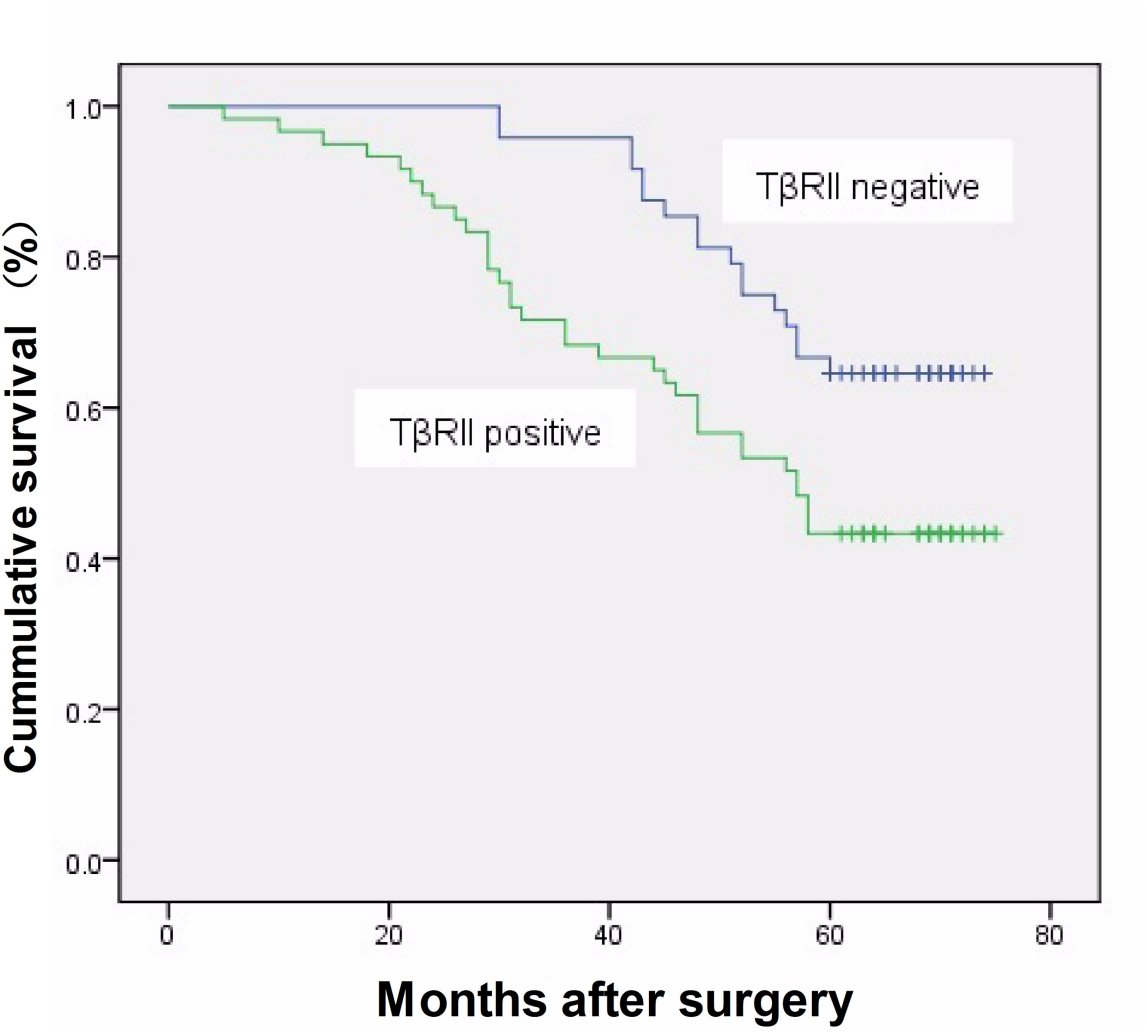
TβRII expression was significantly associated with 5-year disease-free survival (*P* = 0.011).

**Fig 3:**
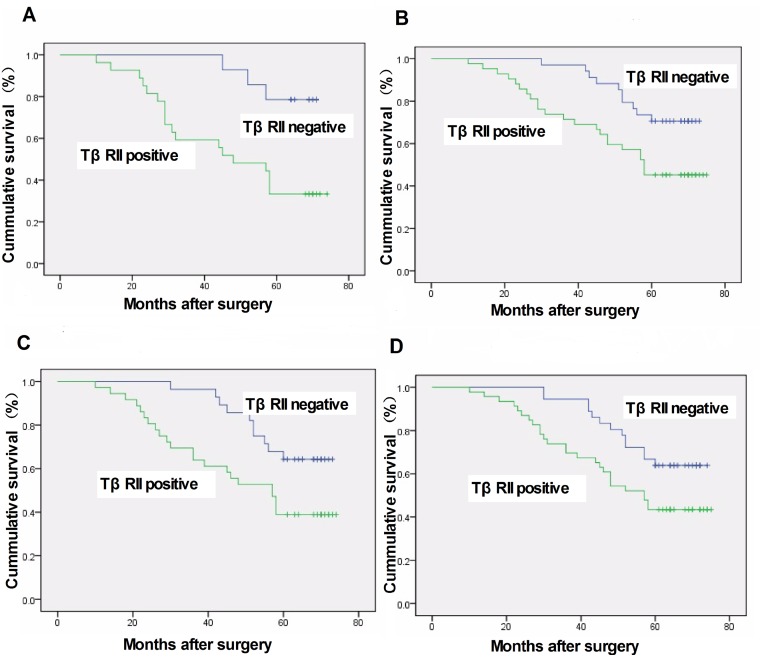
Five-year disease-free survival rate of breast cancer patients stratified according to TβRII expression, other clinicopathological characteristics and immunohistochemical markers. (A) TβRII expression was associated with poorer prognosis in lymph node-positive patients (*P* = 0.006).(B) TβRII expression was associated with shorter 5-year disease-free survival in patients with ER-positive breast cancer (*P* = 0.016).(C) TβRII expression was associated with shorter 5-year disease-free survival in the patients with PR-positive breast cancer (*P* = 0.022).(D) In patients lacking HER2 expression, there was a significant association between TβRII expression and 5-year disease-free survival (*P* = 0.033).

After a median follow-up time of 54.96 months (range, 5–75 months), 51 women (54.4%) had relapsed and died, and 5-year DFS rate was 45.6%. Patients with stage I or II disease had a significantly longer DFS rate than those with stage III disease (50.0% *vs*. 60.94% *vs*. *21*.*43*%; *P* = 0.026). Univariate analysis showed that elevated TβRII expression was associated with poor DFS of breast cancer (*P* = 0.028). However, there was no association of survival rates and tumor size, lymph node status, and the expression of ER, PR, and HER-2. Survival analysis demonstrated that TβRII was associated with poor DFS (*P* = 0.011, log rank test; Fig 2). Subgroup analysis revealed that TβRII expression was associated with a shorter DFS rate in patients with lymph node metastasis, ER-positive, PR-positive, and Her-2-negative tumors (*P* = 0.006, *P* = 0.016, *P* = 0.022, and *P* = 0.033, respectively; log rank test; Fig 3A–3D). Furthermore, multivariate analysis using Cox proportional hazards model revealed that TβRII was an independent prognostic factor for breast cancer.

## Discussion

TGF-β signaling regulates multiple aspects of tumor progression, including the proliferation, apoptosis, and metastasis of tumor cells, as well as the maintenance of tumor-initiating cells; TGF-β has either a tumor suppressing or tumor promoting function depending on cellular context [15].

In the present study, our data demonstrated that positive TβRII expression was associated with lymph node metastasis and increasing clinical stage. This observation is consistent with the concept that in late-stage human cancers, the TGF-β signaling pathway functions as a tumor-promoter, which is associated with an aggressive tumor phenotype and metastasis [22,23]. Previous works suggests that decreased TβRII is associated with an increased risk of developing invasive breast cancer, and that TβRII is a marker of poor prognosis [7,11]. In the TGF-β signaling pathways, TβRII is critical for transcription. Tumor cells are less sensitivive to TGF-β-mediated growth inhibitory responses upon TβRII down-regulation [24]. In contrast, our study showed that higher TβRII expression was correlated with poorer survival outcome. There may be some possible reasons behind these discrepant results, such as the ethnicity of the patient population, the sample size, and the varying clinicopathological features including clinical stage, tumor histological type, grade, and others. However, perhaps more importantly,the methods of evaluating TβRII expression by immunohistochemistry are different in various studies. Perhaps, the additional methods to evaluate the TβRII expression can provide more information for the discrepancy from different studies. So, the improved experimental design must be needed for the further research.

Several studies have concentrated more on the relationship between ER and TGF-β signaling. It is reported that ER and ER-α suppressed breast cancer metastasis by inhibiting TGF-β signaling [25]. ER-negative tumors that express TβRII or demonstrate a TGF-β response transcript signature have been associated with reduced overall survival [26]. High expression of TβRII is associated with ER positivity [19]. In our study, after the subgroup analysis, ER positive tumors that expressed TβRII was associated with shorter DFS.

However there are still some other components involved in the TGF-β signaling pathway, including TGF-β receptors I, nuclear phosphorylated-Smad2 and Smad4. Previous study shown that the presence of phosphorylated-Smad2 (p-Smad2, indicative of active canonical TGF-β signaling) was associated with positive nodal status [20] and it was reported that high expression of Smad4 is associated with a favorable prognosis [19]. These TβRII downstream targets were also associated with the prognosis of breast cancer. In further studies, these biomarkers should be combined to explore the interactions and provide more information about the TGF-β signaling pathway.

In summary, our results demonstrate that high expression of TβRII in breast cancer cells may be a prognostic marker for breast cancer patients and the deep research is needed to provide useful information for evaluating the effect of TGF-β in the development of breast cancer.

## Supporting Information

S1 File The relationship between TβRII expression and the status of lymphnode metastasis was analysed by the Chi-squared test. (PDF)
