# Supplementary material for: Clinical Implications of TβRII Expression in Breast Cancer
Source: PLoS One. 2015 Nov 9;10(11):e0141412. doi: 10.1371/journal.pone.0141412 (PMC4638357; doi:10.1371/journal.pone.0141412)
Supplement: S1 File — (PDF) [file pone.0141412.s001.pdf]

```
CROSSTABS
  /TABLES=LN BY TGFBRII
  /FORMAT=AVALUE TABLES
  /STATISTICS=CHISQ
  /CELLS=COUNT ROW
  /COUNT ROUND CELL.
```

Crosstabs

Notes

	Output Created	02-一月-2014 19时13分51秒
	Comments	
Input	Data	C:\Users\Administrator\Desktop\TGFβ-RII\spss 完整数据_1.sav
	Active Dataset	数据集1
	Filter	<none>
	Weight	<none>
	Split File	<none>
	N of Rows in Working Data File	125
Missing Value Handling	Definition of Missing	User-defined missing values are treated as missing.
	Cases Used	Statistics for each table are based on all the cases with valid data in the specified range(s) for all variables in each table.
	Syntax	CROSSTABS /TABLES=LN BY TGFBRII /FORMAT=AVALUE TABLES /STATISTICS=CHISQ /CELLS=COUNT ROW /COUNT ROUND CELL.
Resources	Processor Time	0:00:00.000
	Elapsed Time	0:00:00.000
	Dimensions Requested	2
	Cells Available	174762

[数据集1] C:\Users\Administrator\Desktop\TGFβ-RII\spss 完整数据_1.sav

Case Processing Summary

	Cases					
	Valid		Missing		Total	
	N	Percent	N	Percent	N	Percent
LN * TGFB-RII	108	86.4%	17	13.6%	125	100.0%

LN * TGFB-RII Crosstabulation

			TGFB-RII		
			negative	positive	Total
LN	阴性	Count	33	29	62
		% within LN	53.2%	46.8%	100.0%
	阳性	Count	15	31	46
		% within LN	32.6%	67.4%	100.0%
	Total	Count	48	60	108
		% within LN	44.4%	55.6%	100.0%

Chi-Square Tests

	Value	df	Asymp. Sig. (2-sided)	Exact Sig. (2-sided)	Exact Sig. (1-sided)
Pearson Chi-Square	4.546[a]	1	.033		
Continuity Correction[b]	3.749	1	.053		
Likelihood Ratio	4.605	1	.032		
Fisher's Exact Test				.050	.026
Linear-by-Linear Association	4.504	1	.034		
N of Valid Cases	108				

a. 0 cells (.0%) have expected count less than 5. The minimum expected count is 20.44.

b. Computed only for a 2x2 table
